# Strain engraftment competition and functional augmentation in a multi-donor fecal microbiota transplantation trial for obesity

**DOI:** 10.1186/s40168-021-01060-7

**Published:** 2021-05-13

**Authors:** Brooke C. Wilson, Tommi Vatanen, Thilini N. Jayasinghe, Karen S. W. Leong, José G. B. Derraik, Benjamin B. Albert, Valentina Chiavaroli, Darren M. Svirskis, Kathryn L. Beck, Cathryn A. Conlon, Yannan Jiang, William Schierding, David J. Holland, Wayne S. Cutfield, Justin M. O’Sullivan

**Affiliations:** 1 The Liggins Institute, University of Auckland, Auckland, New Zealand; 2 The Broad Institute of MIT and Harvard, Cambridge, MA USA; 3 A Better Start–National Science Challenge, Auckland, New Zealand; 4 School of Pharmacy, Faculty of Medical and Health Sciences, University of Auckland, Auckland, New Zealand; 5 School of Sport, Exercise and Nutrition, College of Health, Massey University, Auckland, New Zealand; 6 Department of Statistics, University of Auckland, Auckland, New Zealand; 7 Department of Infectious Diseases, Counties Manukau District Health Board, Auckland, New Zealand

## Abstract

**Background:**

Donor selection is an important factor influencing the engraftment and efficacy of fecal microbiota transplantation (FMT) for complex conditions associated with microbial dysbiosis. However, the degree, variation, and stability of strain engraftment have not yet been assessed in the context of multiple donors.

**Methods:**

We conducted a double-blinded randomized control trial of FMT in 87 adolescents with obesity. Participants were randomized to receive multi-donor FMT (capsules containing the fecal microbiota of four sex-matched lean donors) or placebo (saline capsules). Following a bowel cleanse, participants ingested a total of 28 capsules over two consecutive days. Capsules from individual donors and participant stool samples collected at baseline, 6, 12, and 26 weeks post-treatment were analyzed by shotgun metagenomic sequencing allowing us to track bacterial strain engraftment and its functional implications on recipients’ gut microbiomes.

**Results:**

Multi-donor FMT sustainably altered the structure and the function of the gut microbiome. In what was effectively a microbiome competition experiment, we discovered that two donor microbiomes (one female, one male) dominated strain engraftment and were characterized by high microbial diversity and a high *Prevotella* to *Bacteroides* (P/B) ratio. Engrafted strains led to enterotype-level shifts in community composition and provided genes that altered the metabolic potential of the community. Despite our attempts to standardize FMT dose and origin, FMT recipients varied widely in their engraftment of donor strains.

**Conclusion:**

Our study provides evidence for the existence of FMT super-donors whose microbiomes are highly effective at engrafting in the recipient gut. Dominant engrafting male and female donor microbiomes harbored diverse microbial species and genes and were characterized by a high P/B ratio. Yet, the high variability of strain engraftment among FMT recipients suggests the host environment also plays a critical role in mediating FMT receptivity.

**Trial registration:**

The Gut Bugs trial was registered with the Australian New Zealand Clinical Trials Registry (ACTRN12615001351505).

**Trial protocol:**

The trial protocol is available at https://bmjopen.bmj.com/content/9/4/e026174.

**Video Abstract**

**Supplementary Information:**

The online version contains supplementary material available at 10.1186/s40168-021-01060-7.

## Background

Fecal microbiota transplantation (FMT) is currently being investigated for its efficacy in treating a variety of chronic disorders in which the gut microbiome is presumably implicated. Among these, obesity and metabolic disorders have been a key focus, given their global health burden and lack of effective treatment options [1]. Pioneering experiments in mice revealed that obese and lean phenotypes could be transferred through the fecal microbiota of human donors [2–4]. Clinical trials of FMT have been comparatively less impressive with respect to weight loss in individuals with obesity [5–9]. Nonetheless, FMT has been shown to transiently improve insulin sensitivity in some individuals [8, 9] and alter fat distribution in others [10]. While these studies point to a complex relationship between the human gut microbiota and metabolic health, the mechanisms and levels of strain engraftment required to elicit these effects remain poorly characterized.

Efficient engraftment of donor strains is likely a prerequisite for functional alteration of the host’s gut microbiome [11]. Several studies trialing FMT for inflammatory bowel disease (IBD) have reported greater clinical response in recipients whose gut microbiomes are more receptive to FMT engraftment [12–16]. Similarly, the composition of the donor’s gut microbiota has also been shown to influence FMT outcomes [12, 13, 17]. The idea that some stool is therapeutically better than others led to the concept of “super-donors” [11] and has prompted researchers to trial multi-donor FMT approaches [18] or employ more rationalized donor selection based on perceived microbiota fitness [19]. Multi-donor FMT involves the administration of fecal microbiota from multiple donors, with the goal of homogenizing donor-specific effects and increasing microbial diversity [20].

Replacing a disease-associated microbiome with a one-off dose of donor microbiota is not trivial and presumably involves a variety of competitive interactions between the endogenous and exogenous communities, restrained within the niche environment supplied by the host. Strain-resolved metagenomics have been instrumental in improving our understanding of FMT engraftment, revealing that donor strains can stably coexist with recipient’s endogenous strains for months after FMT [21, 22]. However, the picture is far from complete, and the dynamics and functional implications of strain engraftment in the context of multiple donor sources are yet to be investigated.

To address these gaps, we metagenomically profiled stool samples from healthy, lean donors and adolescents with obesity participating in a double-blinded randomized-controlled trial for FMT. Fecal microbiota was harvested from multiple donors and administered over two consecutive days in a capsule form that enabled competition between the individual microbiomes. Recipients’ gut microbiomes were tracked for up to 6 months post-treatment. Specifically, we sought to quantify the degree of donor strain engraftment, uncover any donor-specific trends, track the persistence of donor strains, and identify any functional effects of strain engraftment on the host’s microbial community.

## Methods

### Study design and participant information

The Gut Bugs trial was a double-blinded, randomized, placebo-controlled trial that tested the efficacy of FMT capsules to treat adolescents with obesity [23]. This study was approved by the Northern A Health and Disability Ethics Committee on 8th November 2016 (16/NTA/172). A total of 87 post-pubertal adolescents with obesity participated in the trial (51 females, 36 males, aged 14–18 years, BMI 37.7 ± 5.3 kg/m^2^, total body fat 47.5 ± 5.6%). Nine donors (4 females, 5 males, aged 19–27 years, BMI 22.7 ± 1.9 kg/m^2^, total body fat 18.4 ± 3.2%) were selected following extensive health and pathogen screening as documented in the published trial protocol [23].

Recipients were stratified by sex and randomized 1:1 to receive 28 acid-resistant capsules over two consecutive days containing either the active treatment (multi-donor FMT) or placebo (saline solution). The fecal microbiota used for FMT capsules were derived from four healthy same-sex lean donors. To standardize treatment, the same donors were used for the entire course of the study with the exception of one male donor, DM05, who was replaced after the first male treatment round with DM12 due to illness.

The day before treatment, recipients ingested a GlycoPrep-C solution (Fresenius Kabi, New Zealand) to cleanse the bowel and reduce the endogenous microbial load. Participants were instructed to maintain their usual diet and physical activity patterns throughout the trial. Clinical assessments were conducted at baseline, 6 weeks, 12 weeks, and 26 weeks post-treatment [23].

### Fecal microbiota transplantation procedure

Fecal microbiota was harvested from fresh donor stool and double encapsulated using a modification of a validated protocol [24]. Capsules were prepared using acid-resistant DR Caps^TM^ (Capsugel, USA) which are specifically designed to release their contents in the proximal bowel. Each capsule contained 0.25 g of microbiota suspended in 0.5 ml cryoprotective saline solution (0.9% NaCl, 15% glycerol). Placebo capsules were visually indistinguishable and contained 0.5 ml of sterile saline solution (0.9% NaCl, 15% glycerol). Capsules were stored at −80°C for approximately 1 week before administration. The capsule bacterial load was assessed by quantitative PCR using universal 16S primers (Fwd: 5′-GGTGAATACGTTCCCGG-3′; Rev: 5′-TACGGCTACCTTGTTACGACTT-3′). We did not observe any significant differences in FMT capsule bacterial load between donors (Suppl. Fig. 1).

Due to the nature of recruitment, participants were treated in batches, with fresh FMT capsules prepared for each treatment batch. In total, there were 8 female and 6 male treatment batches. Following a bowel cleanse and overnight fast, participants received their allocated capsules. FMT recipients received 7 capsules from each of the 4 same-sex donor, for a combined dose of 28 capsules: 16 capsules on the first day (4 capsules/donor) and 12 capsules the following day (3 capsules/donor). Capsules were swallowed with a glass of water under clinical supervision.

### Sample collection and processing

Fresh stool samples were collected on site from participants at baseline (prior to bowel cleansing and treatment), as well as at 6 weeks, 12 weeks, and 26 weeks post-treatment. A 200 mg aliquot was taken from the middle section of the stool and transferred to a 2-ml LoBind DNA tube for temporary storage at −80°C. In addition, FMT capsules from each batch of donations were reserved for microbiome assessment. Nucleic acid extraction was performed within 5 days of stool collection using a modified protocol of the AllPrep DNA/RNA Mini Kit (Qiagen, USA) [25]. Firstly, stool aliquots were incubated in 100 μl of lysis buffer (30 mM Tris-HCl, 1 mM EDTA, 15 mg/ml lysozyme) for 10 min at room temperature with regular agitation. Samples were then mixed with 1.2 ml RLT plus buffer (Qiagen, USA), 12 μl beta-mercaptoethanol (Sigma-Aldrich, USA), and 1 ml of acid-washed glass beads (≤106 μm, −140 US sieve; Sigma Aldrich, USA) and shaken vigorously at 30 Hz for 10 min on a TissueLyser II (Qiagen, USA). The homogenate was then passed through a QIAshredder spin column (Qiagen, USA), before continuing on with the standard AllPrep DNA/RNA Mini Kit protocol (Qiagen, USA) eluting in 100 μl of EB buffer. DNA purity was assessed using a NanoPhotometer N60 (Implen GmbH, Germany) and quantified by Qubit dsDNA Broad Range Assay (Thermo Fisher Scientific, USA).

### Metagenomic library preparation and sequencing

A total of 381 stool samples from recipients and donors were analyzed by shotgun metagenomic sequencing. Metagenomic sequencing libraries were prepared using the NEBNext Ultra DNA Library Prep Kit for Illumina (NEB, USA) following the manufacturer’s protocol. In brief, DNA was fragmented by sonication to an average size of 300 bp, and resulting fragments were end-polished, A-tailed, and ligated with sequence adaptors. Following PCR amplification, DNA fragments were purified (AMPure SP system, Beckman Coulter, USA), assessed for size distribution (Agilent2100 Bioanalyzer, USA), and quantified by real-time PCR. Clusters were generated using the cBot Cluster Generation System, and sequencing was performed on an Illumina NovaSeq6000 platform, generating an average of 23 million reads per sample (150 bp paired-end reads). Raw sequencing files were processed with bioBakery workflows using docker images available at http://huttenhower.sph.harvard.edu/biobakery_workflows. Quality control and pre-processing steps involved removal of adaptor sequences using Trim Galore! followed by removal of low-quality reads and human sequences with KneadData. Post-processed metagenomic sequencing files and accompanying metadata are deposited in NCBI’s SRA database (BioProject PRJNA637785).

### Taxonomic profiling

Species-level taxonomic profiles were generated by MetaPhlAn2 v2.7.7 [26]. Metaphlan2 uses read coverage of clade-specific marker genes to estimate the relative abundance of taxonomic clades present within a sample. Taxonomic profiles included bacteria, archaea, viruses, and eukaryotic microbes. Shannon’s diversity index was used to estimate alpha diversity, and Bray-Curtis dissimilarity was used to estimate beta diversity at the bacterial species level.

Differences in the overall structure of the gut microbiome, based on Bray-Curtis dissimilarities, were assessed by permutational multivariate analysis of variance (PERMANOVA) using the adonis2 function in the vegan R package [27]. The effect of treatment group was assessed cross-sectionally at each time point, with marginal adjustments for sequence batch, sex, age, ethnicity, and antibiotic usage (10,000 permutations). Due to the small sample size, we did not adjust for diet. However, recipients were asked to maintain their normal diet throughout the study period. Nominal *p*-values from PERMANOVA were adjusted for multiple testing using Benjamini-Hochberg procedure to obtain *q*-values, and results with *q* < 0.1 were considered statistically significant.

Associations between individual species and metadata were examined using general linear models as implemented in the MaAsLin2 R package [28]. Species relative abundance profiles were log-transformed, and species had to be present in at least 10% of samples to be included in analyses. For assessing the effect of treatment from baseline to week 6 on species relative abundance, time point was used as a fixed effect variable, with participant ID added as a random effect, including each treatment group in turn (i.e., FMT and placebo profiles run separately). Differential species associated with FMT were visualized by pheatmap [29], with manual clustering according to the significance level and direction of association.

### Strain inference and engraftment analysis

Profiling of the dominant strain of a given species was achieved by SNP-based haplotyping using StrainPhlAn [30], requiring a minimum coverage of 5 bases for SNP calling (min_read_depth 5), and adding the option “--relaxed_parameters3”. To estimate phylogenetic relationships between conspecific strains from different individuals, DNA similarity distances were calculated for SNP haplotypes using the Jukes and Cantor (JC69) model, as implemented in the phangorn R package [31]. An initial tree was constructed using Unweighted Pair Group Method with Arithmetic Mean (UPGMA) hierarchical clustering, which was optimized by maximum likelihood estimation using the Kimura model (K80). Phylogenetic trees were visualized using the ggtree R package [32].

To systematically quantify strain engraftment events for all profiled species, we employed the same method used by Ferretti et al. [33]. In brief, DNA distances were normalized by the median distance across all pairwise comparisons of a given species enabling the varying degrees of strain diversity across species to be taken into account. By comparing the distribution of normalized DNA distances for strains from the same donor (i.e., intra-pair) and between donor and recipient strains (i.e., inter-pair), we noted two distinct peaks (Fig. 3b); the peak close to 0 represented near-identical strains. Based on this plot, we selected a conservative normalized DNA distance threshold of 0.2 such that any strains with higher similarity were deemed a strain match (Fig. 3b).

### Functional profiling

Functional profiling was performed by HUMAnN2 v0.11.2 [34], which involved mapping post-processed reads against the pangenomes of detected species, allowing read-count-based quantification of the microbial gene families present within each sample. Identified gene families were further mapped to the MetaCyc database to provide quantification of metabolic pathways. Both gene family and pathway profiles were stratified by contributing organisms. For each sample, gene richness was calculated by counting the number of unique gene families present. When comparing gene richness of donors and participants at baseline, multiple samples from each donor, corresponding to each donation batch, were averaged. Associations between individual pathways and treatment were assessed cross-sectionally for each time point with general linear models using MaAsLin2 [28]. Gene families belonging to enriched pathways associated with FMT were extracted by unpacking the reaction components of the pathway. For each enriched pathway, we looked for examples of gene families that were not present within recipients at baseline, but were present 6 weeks post-treatment, and were also present in any one of the contributing donors. To be confident in calling gene presence, the species genome it belonged to was required to be present within a sample at sufficient coverage, such that its absence could not be explained by incomplete genome representation. Inspecting the number of genes for a given species within a sample by the median copies per million (CPM) of those genes enabled us to select a conservative universal genome coverage threshold (median CPM >4), above which we could confidently call gene presence.

## Results

### Overview of the Gut Bugs trial

**Fig. 1:**
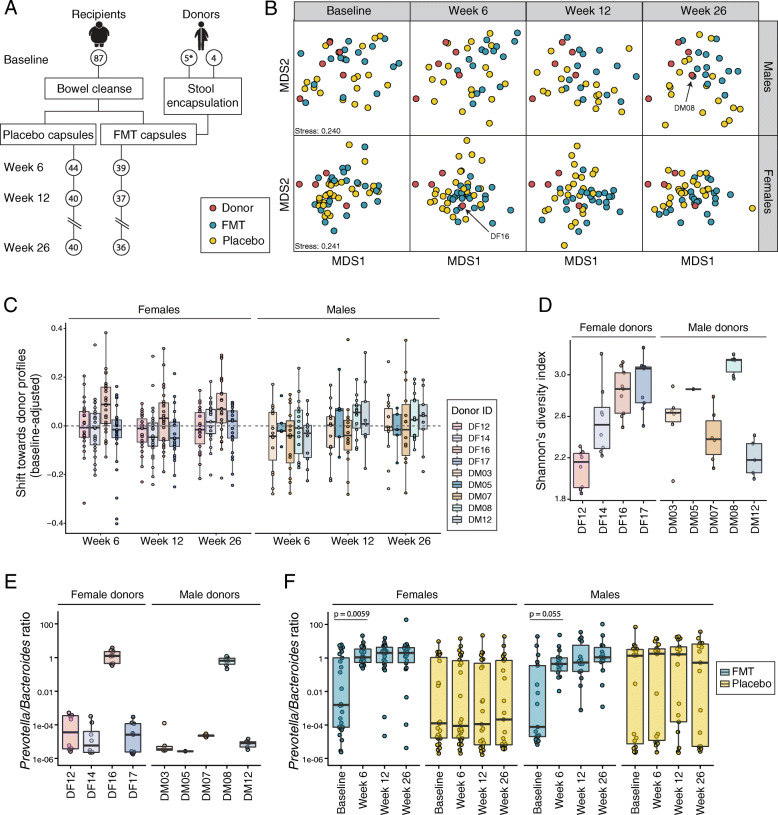
FMT led to prominent shifts in the gut microbiome composition towards particular donors. **a** Design of the Gut Bugs trial. Circles represent stool sample collection time points with corresponding participant numbers indicated. *One male donor was replaced during the trial; hence, 5 male donors were recruited. **b** Multidimensional scaling plots based on species-level Bray-Curtis dissimilarities, subset by sex and surveyed time point. Multiple samples from each donor, corresponding to each donation batch, were averaged to generate a composite donor profile. **c** Shifts in similarity of FMT recipients’ fecal metagenome to each contributing donor after adjusting for baseline similarity. **d** Alpha diversity of the gut microbiome of donors as measured by Shannon’s diversity index. Multiple points correspond to separate donations. **e**, **f** *Prevotella*/*Bacteroides* ratio of the gut microbiome of donors (**e**) and FMT and placebo recipients (**f**). Differences from baseline to week 6 were measured by Wilcoxon signed-rank test

The Gut Bugs trial was a double-blinded, randomized-controlled FMT trial that recruited 87 adolescents with obesity (Fig. 1a). FMT recipients received a total of 28 capsules containing 7g of concentrated fecal microbiota derived from four sex-matched lean donors (1.75g microbiota per donor). Placebo recipients received 28 capsules containing a saline solution. All recipients underwent a bowel cleanse the day before treatment. Capsules were ingested across two clinic sessions, one day apart. Clinical assessments and stool samples were collected at baseline (before the bowel cleanse) and at 6, 12, and 26 weeks post-treatment. FMT did not lead to a reduction in body weight or BMI. However, FMT recipients exhibited a reduction in android-to-gynoid fat ratio at all post-treatment time points, an effect that was more marked among females. Furthermore, FMT resulted in a 4.5-fold reduction in the prevalence of metabolic syndrome at week 26 (for full trial results see [10]).

### Multi-donor FMT altered gut microbiome composition long-term

A total of 381 stool samples from recipients and donors were analyzed by shotgun metagenomic sequencing at a mean sequencing depth of 23 million reads/sample (mean 6.9 Gb/sample). To assess the impact of FMT on gut microbiome composition, we generated a Bray-Curtis dissimilarity matrix for all fecal metagenome samples and performed a series of cross-sectional PERMANOVA tests. After adjusting for known microbiome confounders (i.e., age, sex, ethnicity, and sequence batch), treatment group was found to have a significant effect on the gut microbiome composition at week 6 and week 12 accounting for 2.7% and 2.9% of the variance, respectively (FDR corrected *q* < 0.1, Suppl. Table 1). This shift in overall community composition was also accompanied by a temporary increase in alpha diversity at week 6, specifically within female FMT recipients (Wilcoxon signed-rank test, *p* = 0.0043, Suppl. Fig. 2). Female placebo recipients also exhibited a temporary increase in alpha diversity from baseline to week 12 (Wilcoxon signed-rank test, *p* = 0.014, Suppl. Fig. 2). Across all post-treatment time points, both male and female FMT recipients had a significantly higher dissimilarity to their baseline sample compared to placebo recipients (Wilcoxon rank-sum test, *p* < 0.005). This indicated that a one-off dose of FMT capsules was capable of inducing sustained changes in the gut microbiome for up to 26 weeks post-treatment, above the spontaneous drift observed over the same time period within placebo recipients.

### Gut microbiomes of female FMT recipients clustered around one particular donor

To visualize the variation in gut microbiome composition, we performed multidimensional scaling (MDS) on the species-level Bray-Curtis dissimilarities. Stratified by sex and time point, we observed distinct patterns of microbiome shift in response to FMT (Fig. 1b). Strikingly, the gut microbiomes of female FMT recipients clustered around just one of the four contributing donors, particularly at week 6. By contrast, the gut microbiomes of male FMT recipients did not appear to move towards one particular donor. However, we did observe a slight clustering of male FMT recipient samples, suggesting their microbiomes had become more similar to one another post-FMT.

After adjusting for baseline similarity, we confirmed that the gut microbiome of female FMT recipients became more similar to female donor DF16 (Fig. 1c). The shift towards DF16 among FMT recipients was observed at week 6 and week 26, but not at week 12 (Wilcoxon rank-sum test; week 6, *p* = 0.016; week 12, *p* = 0.27; week 26, *p* = 0.0031, Suppl. Table 2). Within male FMT recipients, there was a subtle shift towards male donor DM08, at week 12 and week 26 (Wilcoxon rank-sum test; week 12, *p* = 0.059; week 26, *p* = 0.044, Suppl. Table 2).

DF16 had the second highest alpha diversity among the female donors, while DM08 was the most diverse male donor (Fig. 1d). There was a positive correlation between the degree of microbiome shift towards a donor and the donors’ alpha diversity (Spearman’s correlation, *ρ* = 0.25, *p* = 0.0013).

### FMT transitioned gut microbiomes from *Bacteroides* to *Prevotella* dominance

Recent studies have suggested individuals can be stratified into microbial enterotypes according to the ratio of *Prevotella* to *Bacteroides* (P/B ratio) [35, 36]. To investigate whether the observed shifts towards DF16 could be explained by a transition in the P/B ratio, we compared the P/B ratio of donors to those of recipients before and after treatment. As in previous studies [5, 37], we defined P/B ratios above 0.1 as being high. Female donor DF16 and male donor DM08 were the only donors with a high P/B ratio (Fig. 1e). At baseline, recipients displayed variable P/B ratios, with some recipients characterized by high levels of *Prevotella* and others by high levels of *Bacteroides* (Fig. 1f). For placebo recipients, the P/B ratio distribution did not change during the 6-month trial, consistent with the individual’s enterotypes remaining stable. By contrast, among the FMT recipients, almost all individuals with a low P/B ratio at baseline transitioned to a high P/B ratio following FMT (week 6; Wilcoxon signed-rank test, *p* = 0.0011). This shift from *Bacteroides* to *Prevotella* dominance was largely maintained by FMT recipients out to 26 weeks post-treatment (week 26; Wilcoxon signed-rank test, *p* < 0.001, Fig. 1f).

### Dominant donor contributed to species enrichment post-FMT

**Fig. 2:**
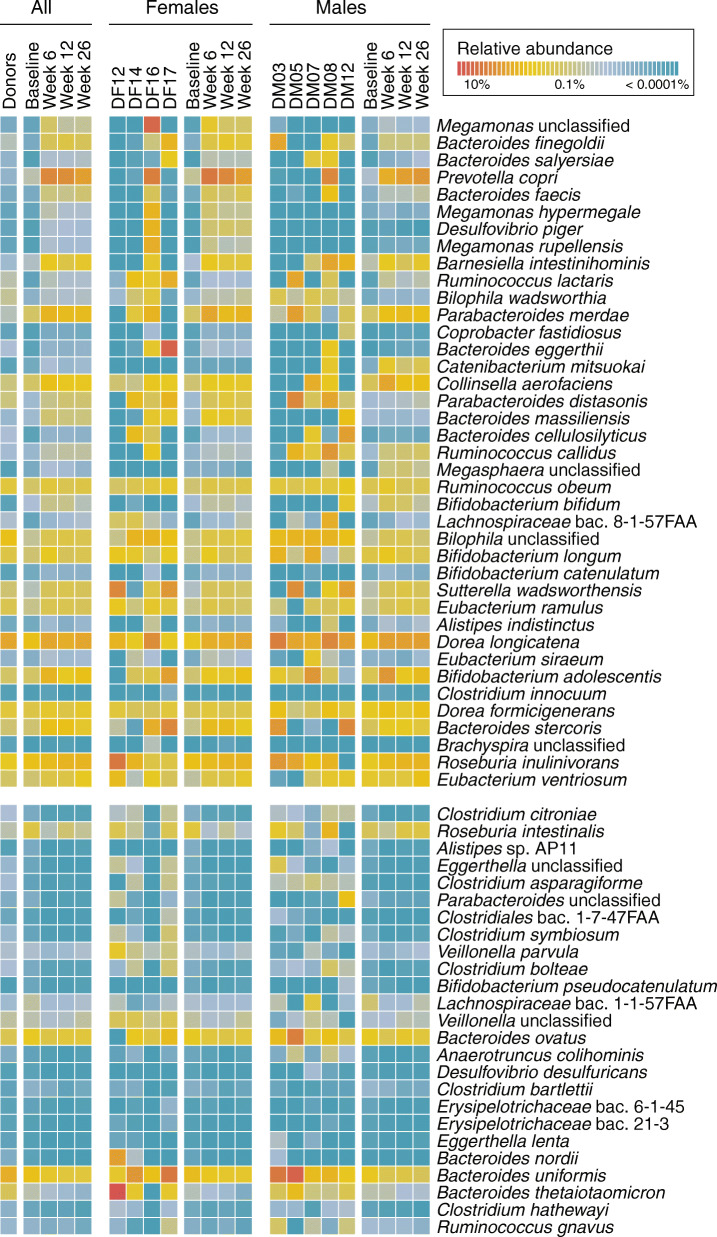
Bacterial species of the gut microbiome whose relative abundance was altered post-FMT. Species are grouped according to whether they were enriched (top panel) or reduced (bottom panel) post-FMT and are listed in order of statistical significance from week 6 onwards (linear model, FDR adjusted *q* < 0.1). Relative abundances were log_10_-transformed with a small pseudo-count (1E-06) added to account for zero abundance values. A relative abundance < 0.0001% signifies that the species did not pass the minimum threshold abundance level for quantification. Each cell represents the mean transformed relative abundance for a specific species according to the grouping variable; “All” combines male and female averages, while “Females” and “Males” allow species abundances to be subset by sex and contributing donors. Placebo recipient profiles are not displayed, as no bacterial species in their gut microbiome were significantly altered throughout the course of the study.

The shift towards DF16 among female FMT recipients and the more subtle shift towards DM08 among males may be partially explained by the P/B ratio transition. However, to gain a broader perspective of donor-dependent effects, we surveyed all detectable species to identify those whose relative abundance was significantly altered post-treatment. Using general linear modeling as implemented in MaAsLin2, we identified 64 bacterial species that were differentially abundant between baseline and post-treatment time points in FMT recipients (FDR adjusted *q* < 0.1). Of these, 39 increased and 25 decreased in relative abundance (Fig. 2). The number of differentially abundant species decreased with time; 51 species were altered at week 6, 45 species at week 12, and 34 species at week 26. Species enrichment varied by sex, likely reflecting the difference in donor material. The most statistically significant species enriched in females included *Megamonas hypermegale*, *Megamonas rupellensis* (and unclassified sequences from the genus *Megamonas*), *Bacteroides finegoldii*, *Bacteroides salyersiae*, *Bacteroides faecis*, *Bacteroides massiliensis*, *Prevotella copri*, *Desulfovibrio piger*, and *Barnesiella intestinihominis*. Whereas in males, the most enriched species included *Catenibacterium mitsuokai, Bacteroides finegoldii, Prevotella copri, Collinsella aerofaciens*, and *Ruminococcus lactaris*. For some species, enrichment could be traced back to one specific donor, predominantly DF16. In fact, many of the species that were unique to, or abundant within DF16, were found to be elevated in female recipients post-FMT (e.g., *Desulfovibrio piger*, *Megamonas rupellensis*, *Megamonas hypermegale*, *Megamonas unclassified*, *Brachyspira unclassified*, and *Bifidobacterium catenulatum*; Suppl. Fig. 3). We also observed a high relative abundance of *Prevotella copri* in DF16 and DM08 donors when compared to the other donors, which likely contributed to its enrichment among male and female FMT recipients.

To assess whether the shift towards donors was driven by the transfer of novel donor taxa, we removed donor species that were not present in FMT recipients at baseline. Repeating our dissimilarity assessments, we observed that the gut microbiomes of female FMT recipients still shifted in similarity towards DF16 despite the absence of her unique species (Wilcoxon rank-sum test, *p* = 0.019). This observation is consistent with donor microbiomes providing recipients with novel taxa that fill available niches, while also signaling for wider changes in the endogenous community structure.

Despite a drift in the gut composition of placebo recipients over the course of the study, no individual species were found to be differentially abundant from baseline. This suggests that the species alterations seen within FMT recipients were specific to the FMT treatment and not caused by the bowel cleanse that preceded treatment, or temporal fluctuations.

### FMT resulted in durable donor strain engraftment

Species identification alone cannot always discriminate between different donor sources, nor can it rule out species acquired from the environment. Hence, to gain a more accurate understanding of FMT engraftment from multiple donors, we extended our taxonomic profiling resolution down to the strain-level. Due to the high level of strain heterogeneity between individuals, finding the same strain from a donor in a post-FMT recipient provides strong evidence of FMT engraftment.

**Fig. 3:**
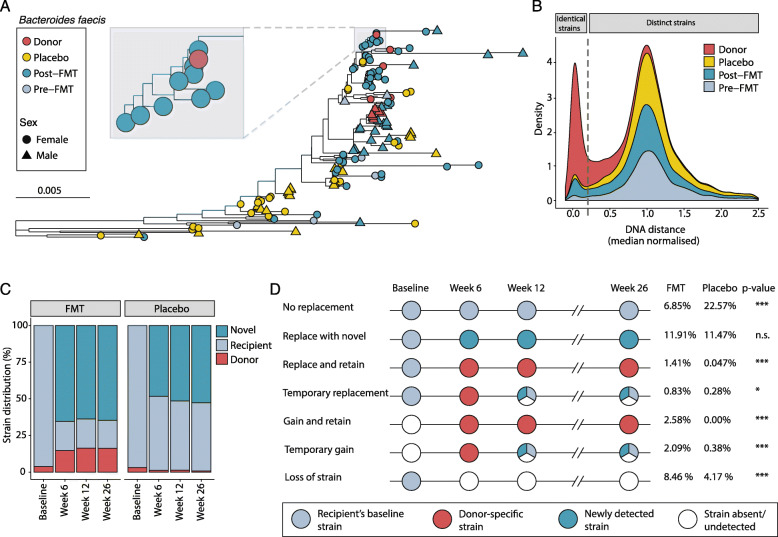
Strain profiling reveals a variety of competition dynamics for conspecific microbial strains. **a** Phylogenetic tree of different *Bacteroides faecis* strains, one of the species enriched post-FMT. *Bacteroides faecis* strains were present in 138 fecal metagenomes as determined by SNP haplotyping. Scale bar signifies difference in sequence similarity between SNP haplotypes. **b** Distribution of median normalized DNA distances for conspecific strain pairs. Recipient strains (pre-FMT, post-FMT, and placebo) were compared against donor strains from the corresponding treatment batch. Because we had multiple stool samples for each donor, we also compared intra-donor strains (plotted in red). This allowed us to set a universal strain threshold of 0.2 median normalized DNA distance for calling identical strains, as indicated by the vertical dashed line. **c** Proportions of strains identified as being either unique to recipient (matching recipient’s baseline strain) or unique to donors (matching any of the contributing donor strains). Strains that were newly detected, or that did not match the recipient’s baseline strain or any contributing donor strains were designated as “Novel”. **d** Proportion of longitudinal strain profiling scenarios by treatment group. Differences between FMT and placebo proportions for each scenario were tested by proportion test with significance denoted by *p < 0.05, ***p < 0.0005, n.s. not significant

The strain profiling approach we used involved mapping reads against a set of species-specific marker genes to generate a single nucleotide polymorphism (SNP) haplotype representing the dominant strain of a given bacterial species within a sample. The SNP haplotypes were used to construct phylogenetic trees and identify donor strain engraftment events. For example, *Bacteroides faecis* strains from FMT recipients (post-FMT) were located in close genetic proximity to donor strains and were clearly separated from their pre-FMT strain (Fig. 3a).

To systematically quantify and monitor strain engraftment events for every species, we selected a genetic strain identity threshold (0.2 median normalized DNA distance), under which strains were considered to be similar enough to constitute a strain match (Fig. 3b). On average, we found that 15% of the strains in the post-FMT microbiome originated from the donors and were retained for up to 26 weeks post-treatment (Fig. 3c). By comparison, placebo recipients consistently harbored ≤ 1% donor-matching strains. Across both treatment groups, there was a high degree of strain instability reflecting the emergence of novel strains that did not match recipients’ baseline strains or any of the donor strains. These novel strains could represent (1) strains acquired from the environment, (2) donor or recipient strains that were below the detection threshold at baseline, or (3) donor or recipient secondary strains that we were unable to identify. Consistent with the degree of species-level alteration induced by FMT, we found a higher proportion of novel strains among FMT recipients at all post-treatment time points (Pearson’s Chi-squared test, *p* < 2.2e^−16^). Collectively, these observation imply that FMT leads to greater fluctuations in strain dominance, which is not solely influenced by donor-engrafting strains, but may also include (a) a higher intrinsic rate of strain fluctuation within the recipient’s endogenous microbiome, (b) an influx of novel donor diversity that facilitates greater engraftment of environmentally acquired strains due to ecosystem rearrangement (consistent with the diversity begets diversity model [38]), or (c) the combined effect of donor and recipient factors.

### Strain profiling over time revealed distinct patterns of donor engraftment

By tracking strain dominance over time, we identified a variety of ecological scenarios which included no replacement, replace with novel, replace and retain, temporary replacement, gain and retain, temporary gain, and loss of strain (Fig. 3d). Combining all profiled species, we found that the proportions of 6 out of 7 scenarios differed between FMT and placebo recipients. For both groups, we observed relatively high rates of dominant strain replacement from baseline. Replacement by a donor strain only accounted for 11% of strain replacement events among FMT recipients suggesting a high degree of strain turnover from uncharacterized sources. Donor strain replacement within FMT recipients tended to be stable out to 26 weeks rather than temporary. As expected, placebo recipients were more likely to retain their dominant strain throughout the course of the study indicating a higher degree of microbiome stability.

Among FMT recipients, the most common species in which the dominant strain was replaced was *Bacteroides stercoris*, whereas recipient strains from *Ruminococcus bromii*, *Bacteroides vulgatus*, *Eubacterium ventriosum*, and *Eubacterium rectale* remained stable, despite also being present in donors. Recipient strains that were present at lower relative abundance were more likely to be replaced than strains that were at higher relative abundance (Wilcoxon rank-sum test, *p* = 0.004, Suppl. Fig. 4A). We also identified cases where recipients had gained a donor strain (either temporarily or long-term) from a species that they did not possess at baseline. This gaining of a new strain occurred more frequently than recipient strain replacement. This result differs from a published observation that conspecific donor strains were more likely to engraft than new species [22]. Finally, among FMT recipients, we discovered a proportionally higher incidence of strain loss without replacement compared to the placebo group. This suggests that the introduction of exogenous strains likely leads to niche replacement and out-competition of both conspecific and heterospecific strains.

### FMT recipients exhibited differential degrees of donor strain engraftment

**Fig. 4:**
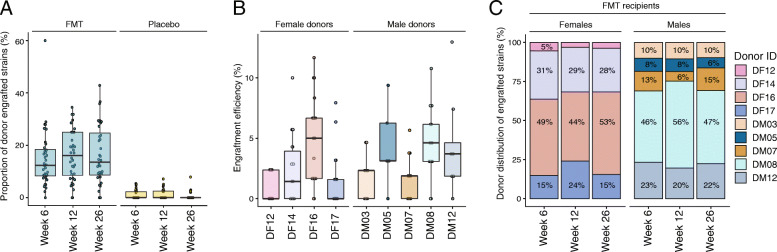
Inter-individual variability in donor strain engraftment. **a** Proportion of donor-engrafted strains in recipients at each post-treatment timepoint. Data points represent recipient fecal metagenome samples. **b** Engraftment efficiency of donors represents the proportion of strains within the donor’s fecal metagenome that engrafted among FMT recipients, detected at week 6. **c** Donor-specific contributions to overall strain engraftment in FMT recipients

The degree of donor strain engraftment varied substantially between FMT recipients (Fig. 4a). While we observed a mean donor-strain engraftment proportion of 15% at week 6, variation among FMT recipients ranged from 0 through to 60%. There was no difference in the mean proportion of donor-strain engraftment between male and female recipients, with both exhibiting high inter-individual variation (Wilcoxon rank-sum test, *p* = 0.34, Suppl. Fig. 4B). Resistance to exogenous donor strain engraftment may reflect the resilience and stability of the endogenous gut community. Microbial diversity is broadly believed to contribute to gut microbiome resilience by providing functional redundancy and colonization resistance [39]. Yet, we found no correlation between recipients’ microbial diversity (Shannon’s diversity, species richness) at baseline and the proportion of donor strain engraftment, suggesting other factors are likely involved.

DF16’s *Bacteroides stercoris* strain engrafted into 11 FMT recipients, and DM03’s *B. stercoris* strain engrafted into 5 FMT recipients. However, in general, recipients contained unique sets of donor-engrafting strains, and the number of recipients was too small to identify consistent engraftment patterns. Notably, the proportion of overall donor strain engraftment, or that of any one donor, did not correlate with changes in any clinical variables (data not shown). Clinical parameters assessed included anthropometric measures, lipid profile, and markers of glucose metabolism. Thus, in the context of obesity, higher levels of donor engraftment were not associated with improvements in any of the clinical outcomes measured.

### Microbially diverse donors contributed more towards strain engraftment

Donors varied considerably in their engraftment efficiency; that is the proportion of their strains that were observed to engraft within the FMT recipients (Kruskal-Wallis rank sum test, females *p* < 0.005, males *p* = 0.022). Donors with higher microbial diversity tended to have higher levels of strain engraftment. Among female donors, DF16 exhibited the highest engraftment efficiency with 4.7% of her strains engrafting in the average FMT recipient (Fig. 4b). By comparison, female donor DF12 had an average engraftment efficiency of just 0.9%. Among the male donors, DM08 had the highest engraftment efficiency of 4.6%, closely followed by DM12 and DM05 both on 4.4% (Fig. 4b). These results were largely consistent with the donor-specific contribution of engrafted strains within FMT recipients (Fig. 4c). Among females at week 6, 49% of donor-engrafted strains belonged to DF16, followed by DF14 on 31%, DF17 on 15%, and DF12 on 5%. A strong donor bias was also apparent in the males, with DM08 contributing to 46% of donor-engrafting strains at week 6, followed by DM12 on 23%, DM07 on 13%, DM03 on 10%, and DM05 on 8%. Notably, DF16 and DM08’s strain engraftment rates did not include *Prevotella* species because strain profiles could not be generated for these species from our data using StrainPhlAn. Therefore, the stronger contributions of DF16 and DM08 were independent of their ability to transfer *Prevotella* strains. Collectively, these strain-level results support the species-level microbiome shifts we observed and provide further evidence that donor microbiomes differ in their engraftment abilities.

### FMT resulted in long-term alterations in the metabolic potential of the gut microbiome

The therapeutic effect of FMT for the treatment of metabolic disorders may lie in the ability of engrafted microbes to transfer beneficial genes that potentiate functional changes at the community level. Therefore, we performed functional profiling using HUMAnN2 [34] to characterize shifts in the functional potential of the microbial gut community in response to FMT.

Previous gut microbiome studies have associated obesity and poor metabolic health with low microbial gene richness [40]. However, we identified no difference in gene richness between our donors and recipients in our study population at baseline (*t*-test, *p* = 0.16). Consistent with the observed variation in alpha diversity (Fig. 1d), we also observed variability in donor gene richness (Suppl. Fig. 5). Female donor DF16 and male donor DM08 exhibited relatively high gene richness in comparison to the other donors, with the exception of male DM07.

**Fig. 5:**
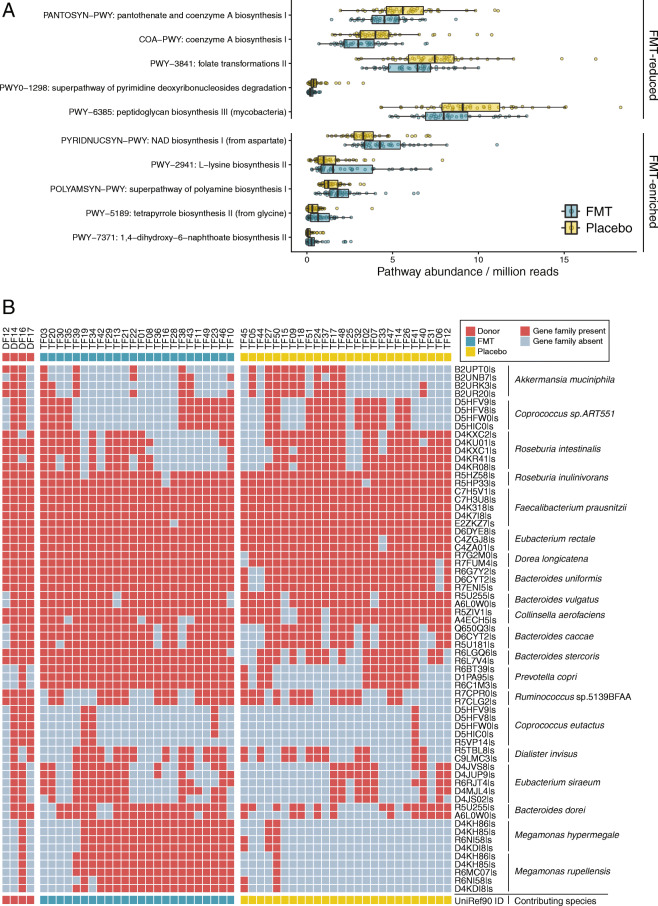
FMT-engrafting strains altered the metabolic capacity of the gut microbiome. **a** Bacterial metabolic pathways in the gut microbiome found to be differentially abundant between FMT and placebo recipients at week 6 (linear model, FDR adjusted q < 0.2). **b** Heatmap displaying UniRef90 gene families belonging to the nicotinamide adenine dinucleotide (NAD) biosynthesis from aspartate pathway that were gained (red cells) by female FMT recipients at week 6 (i.e., were not present at baseline). Placebo recipient data were included to differentiate between environmental gain (gene families likely acquired from common species within the environment) and FMT-specific gain (gene families likely acquired from a donor-engrafting species)

Among FMT recipients, we identified an increase in gene richness from baseline at week 6 (Wilcoxon signed-rank test, *p* < 0.001) and week 26 (Wilcoxon signed-rank test, *p* = 0.018). This effect was particularly notable within females (Suppl. Fig. 5). To determine the impact of this increase in gene richness on community function, we tested for pathways that were differentially abundant between FMT and placebo recipients at each of the surveyed time points. At baseline, there were no treatment-associated pathways. However, at week 6, we identified 10 differentially abundant pathways; 5 that were enriched and 5 that were reduced within FMT recipients (Fig. 5a). The fecal metagenomes of FMT recipients showed a greater potential for nicotinamide adenine dinucleotide (NAD) metabolism, polyamine production, vitamin synthesis (menaquinones and tetrapyrroles), and amino acid metabolism (l-lysine), with concomitant reductions in the potential of a number of pathways, particularly pantothenate and coenzyme A biosynthesis. Subsequent associations at weeks 12 and 26 revealed that the majority of the treatment-associated pathways identified at week 6 were not differentially abundant at later time points. Instead, we identified a further 14 pathways with reduced potential among FMT recipients (Suppl. Table 3). These results confirm that a single FMT dose can cause long-lasting alterations to potential microbial community functions.

### Metabolic pathway enrichment was donor-dependent and linked to strain engraftment

Of the five pathways enriched by FMT at week 6, three were specific to females, and two were specific to males. Female FMT recipients were enriched in the potential for NAD biosynthesis from aspartate, polyamine biosynthesis, and 1,4-dihydroxy-6-naphthoate biosynthesis. By contrast, male FMT recipients were enriched for l-lysine biosynthesis and tetrapyrrole biosynthesis from glycine pathways.

We hypothesized that these sex-specific differences were due to differences in the FMT donor material. To test this, we firstly identified the bacterial species that were responsible for pathway enrichment. Across the three female FMT-enriched pathways, we identified 19 contributing species, four of which were shown to be elevated post-FMT: *Desulfovibrio piger*, *Eubacterium siraeum*, *Megamonas hypermegale*, and *Megamonas rupellensis*. The *Megamonas* species were unique to donor DF16 and contributed genes to all three female FMT-enriched pathways. Similarly, we identified 20 species responsible for the two male FMT-enriched pathways. Three of these species were elevated post-FMT: *Catenibacterium mitsuokai* (unique to male donor DM08)*, Ruminococcus obeum*, and *Sutterella wadsworthensis*.

To determine individual donor contributions to enriched pathways, we tested for genes that had been gained post-FMT that were also present in any of the contributing donors. Across all enriched pathways, we found FMT recipients had gained a higher proportion of these genes compared to placebo recipients, suggesting these had likely been acquired by donor-engrafting species. The majority of the gained genes were traced back to species present within one specific donor. When multiple donors contained the same gene family from the same bacteria, we leveraged dominant strain data to assign the likely donor source. Consistent with these donors having a higher proportion of strain engraftment, we identified the same dominant donors (DF16 and DM08) as contributing proportionately more gene families within the FMT-enriched pathways. For example, female donor DF16 was responsible for the transfer of multiple genes involved in the NAD biosynthesis from aspartate pathway to 18 FMT recipients (Fig. 5b). These genes were present on many of the species identified as being elevated post-FMT, including *Prevotella copri*, and various species from genus *Megamonas*. Importantly, the gain of these genes occurred specifically within FMT recipients, ruling out the possibility that they were naturally acquired from environmental sources. Our ability to track functionally relevant gene transfer from donor to recipient suggests that FMT-related augmentation is largely dependent on which strains the donor provides and which strains engraft within the recipient. Thus, the observed differences in pathway enrichment between males and females suggest that functional alterations induced by FMT are not universal but, rather, specific to the genes present on engrafting strains.

## Discussion

Administration of FMT capsules from multiple donors led to sustained alterations in the structure and function of the gut microbiome in adolescents with obesity. In what was in practice a competition experiment between donor microbiomes, we showed that higher levels of strain engraftment occurred for specific bacterial strains from certain donors. The dominant engrafting female and male microbiomes were characterized by high microbial diversity, high gene richness, and a high *Prevotella* to *Bacteroides* ratio. FMT with these dominant microbiomes resulted in a stable transition towards a *Prevotella*-dominant enterotype in recipients. We unraveled a variety of strain competition dynamics, including replacement of endogenous strains with conspecific donor strains that persisted within their new hosts for at least 6 months. Finally, we tracked the transfer of genes from donor-engrafting strains that led to the enrichment of various metabolic pathways within the recipients’ microbial communities.

Previous studies, based on single donor approaches, have shown that bacterial strains from donors engraft with variable success rates [21, 22]. A larger shift towards the donor, as well as higher levels of FMT engraftment, tends to produce better clinical responses in IBD [12–16]. While we acknowledge that the mechanisms responsible for FMT’s therapeutic effects may extend beyond bacteria [41–43], the key to successful FMT presumably lies in the selection of desirable donors with high engraftment rates. Such donors can arguably be termed “super-donors” if one considers efficient engraftment a prerequisite for clinical improvement [11]. Using a multi-donor approach, we demonstrated that microbiomes from donors with high species diversity and gene richness engraft better than others. This finding is consistent with previous FMT trials for obesity [5] and IBD [12, 17], which also found a positive association between engraftment and microbial diversity of the donor.

We contend that female donor DF16 is an example of a super-donor because of her gut microbiomes’ ability to engraft and augment recipients’ local community structure and function. Remarkably, the microbiome composition of all female FMT recipients shifted towards DF16’s and retained this structure throughout the 6-month trial. Similar donor-polarizing shifts were not observed in male recipients, despite DM08 contributing towards similar levels of strain engraftment and outcompeting the other male donors. We initially hypothesized that the prominent effect of DF16 was due to the transfer of novel taxa that were not present in recipients’ microbiomes at baseline. DF16 had a number of novel species that efficiently transferred, including various *Megamonas* species. Notably, when we removed DF16’s novel taxa from our analysis, we observed that the recipients’ microbiomes were still more similar to hers than those of the other donors. This suggests that, in addition to providing recipients with novel species, engraftment from DF16’s microbiome was also able to alter recipients’ endogenous microbial population structure.

Although we could not assess the clinical efficacy of each individual donor, the use of a single super-donor that induces greater metabolic shifts in recipients’ gut microbiomes may, in theory, lead to greater therapeutic benefits [11]. Using multiple donors may also lead to greater clinical responses compared to single-donor approaches, by providing a diverse variety of strains [18]. Higher diversity increases the likelihood a strain will be able to fill an available niche within the recipient gut. Diversity has also been recently shown to encourage further diversification within the gut as microbial species adapt to their new environment and interact with the resident gut microbiome [38], potentially leading to functional shifts in the community. Despite hypothesizing that greater engraftment would lead to greater clinical response, we found no associations between strain engraftment and clinical improvement across a range of clinical parameters, even when subsetting by donor. These analyses were likely underpowered, particularly given the modest clinical responses observed in the trial [10]. We contend that clinical response to donor engraftment would be better assessed in FMT trials for treatment of more severe forms of dysbiosis (e.g., IBD) where the clinical effect is likely to be more marked.

Recipients were frequently colonized by strains from multiple donors that were largely retained long-term. These results are particularly encouraging given recipients were not asked to change their diet or lifestyle during the course of the study [23]. We did, however, observe highly variable levels of engraftment among FMT recipients despite receiving the same dose of donor material. Although the same set of donors was used throughout the study, we cannot rule out the possibility that there may have been slight variations in microbiota composition between capsule batches. Due to the variable nature of stool, this is an inherent limitation with FMT studies. Recipient variability might also reflect compatibility issues with the donor’s microbiota or higher resilience of the endogenous gut microbiome in some individuals [22]. Receptivity to donor strains was not related to the microbial diversity of the recipient at baseline. This suggests that other selection pressures, such as the recipient’s diet or immune response, may have played a larger role in determining the colonization success of exogenous donor strains.

*Prevotella copri* was found to consistently transfer and stably colonize FMT recipients. Although formal testing of enterotypes was not performed, engraftment of *Prevotella copri* increased the P/B ratio of FMT recipients, effectively switching the recipients’ microbial enterotype. A *Prevotella*-type signature has previously been shown to be more favorable for weight loss in the context of a high-fiber diet [37, 44]. However, microbial enterotypes are relatively stable once established and may not be switched simply by consuming more fiber over a 6-month period [36]. Our results suggest FMT is a highly effective strategy for switching from a *Bacteroides* to a *Prevotella*-type signature. A similar transition from *Bacteroides* to *Prevotella* dominance has been reported before in a multi-donor FMT study for ulcerative colitis which involved regular enema-based administration of pooled fecal material from multiple donors [18]. Unlike our study design, their stool pooling approach was not standardized across batches, with varying numbers and combinations of donors which prevented them from tracing the engraftment of *Prevotella* species to any one particular donor. In our study population, this transition was attributed to the presence of just one *Prevotella*-dominant donor within the multi-donor pool. This suggests that *Prevotella* strains from these donors were able to outcompete the *Bacteroides* strains from the recipients as well as those from the three other donors.

Interestingly, the *Prevotella-*dominant donors we identified were also the most effective donors for overall strain engraftment. Whether this association was causative or not remains unclear. A similar observation was recently reported by a small FMT pilot trial for obesity where the most effective donor for engraftment was also characterized by a high P/B ratio and consistently transferred *Prevotella* species shifting recipient enterotypes [5]. Based on these collective observations, we suggest that future FMT trials for obesity focus on selecting donors with a high P/B ratio, and couple FMT treatment with a high-fiber diet. This may result in maximal donor strain engraftment and help transition individuals towards a microbiome profile that is more susceptible to weight loss.

We demonstrated functional shifts in the metabolic potential of the microbial community following FMT. Importantly, we showed that alterations in microbial metabolic pathways were linked to strain engraftment and newly obtained genes from donor microbiomes, particularly those from the dominant donors. Among the altered microbial pathways, we observed an increased biosynthesis potential for a number of bioactive molecules, including NAD, polyamines, and vitamin precursors, which have previously been implicated in metabolic disorders [45, 46]. NAD, for example, is known to act as an energy sensor and is intricately involved in energy regulation. Low NAD levels are a characteristic of obesity [46, 47], and attempts to boost levels by administering NAD precursors improved weight regulation, glycaemic control, and liver function in mice [48–50]. FMT may thus represent a novel strategy to increase NAD levels via microbial biosynthesis in the gut. However, because NAD levels were not measured in our study, we cannot confirm whether enrichment of this pathway actually led to increased NAD production. Future FMT characterization studies would subsequently benefit from monitoring metabolite production to validate genetic associations and improve our mechanistic understanding of microbiota transfer.

## Conclusion

In conclusion, our study provides further evidence for the existence of FMT super-donors. Dominant engrafting male and female donor microbiomes harbored diverse microbial species and genes, and were characterized by a high P/B ratio. Pre-screening donor microbiomes for these characteristics may help improve donor strain engraftment and elicit greater change in microbial community function. However, donor selection is just one piece of the puzzle. The high variability of strain engraftment among FMT recipients suggests that undetermined host factors still play a significant role in mediating FMT receptivity. Future experiments should focus on identifying the host components that moderate strain engraftment and their interactions with phenotype.

## Supplementary Information

**Additional file 1.** Supplementary Figures

**Additional file 2.** Supplementary Table 1

**Additional file 3.** Supplementary Table 2

**Additional file 4.** Supplementary Table 3

## Data Availability

Post-processed metagenomic sequencing files and accompanying metadata are deposited in NCBI’s SRA database (BioProject PRJNA637785). The complete data processing script is available at https://github.com/brookewilson/gutbugs_microbiome/. BioBakery output tables (KneadData read count table, MetaPhlAn2 relative abundance table, HUMAnN2 pathway abundance table) and the StrainPhlAn SNP haplotype files are available at Figshare (10.17608/k6.auckland.13031681).

## Abbreviations

BMI: Body mass index
CPM: Copies per million
FDR: False discovery rate
FMT: Fecal microbiota transplantation
IBD: Inflammatory bowel disease
NAD: Nicotinamide adenine dinucleotide
P/B ratio: *Prevotella* to *Bacteroides* ratio
PCR: Polymerase chain reaction
PERMANOVA: Permutational multivariate analysis of variance
SNP: Single nucleotide polymorphism
