# Supplementary material for: Strain engraftment competition and functional augmentation in a multi-donor fecal microbiota transplantation trial for obesity
Source: Microbiome. 2021 May 13;9:107. doi: 10.1186/s40168-021-01060-7 (PMC8120839; doi:10.1186/s40168-021-01060-7)
Supplement: Supplementary file 2 — Additional file 1. Supplementary Figures [file 40168_2021_1060_MOESM2_ESM.pdf]

SUPPLEMENTARY FIGURES

Supplementary Figure 1. Bacterial load of FMT capsules by donor.

Supplementary Figure 2. Microbial diversity, as measured by Shannon's diversity index, of donors' and recipients' gut microbiomes.

Supplementary Figure 3. Venn diagrams displaying the number of bacterial species in the gut microbiome that were unique to or shared between **(A)** female donors and **(B)** male donors.

Supplementary Figure 4. Strain engraftment dynamics.

Supplementary Figure 5. Gene richness of donors' and recipients' gut microbiomes.

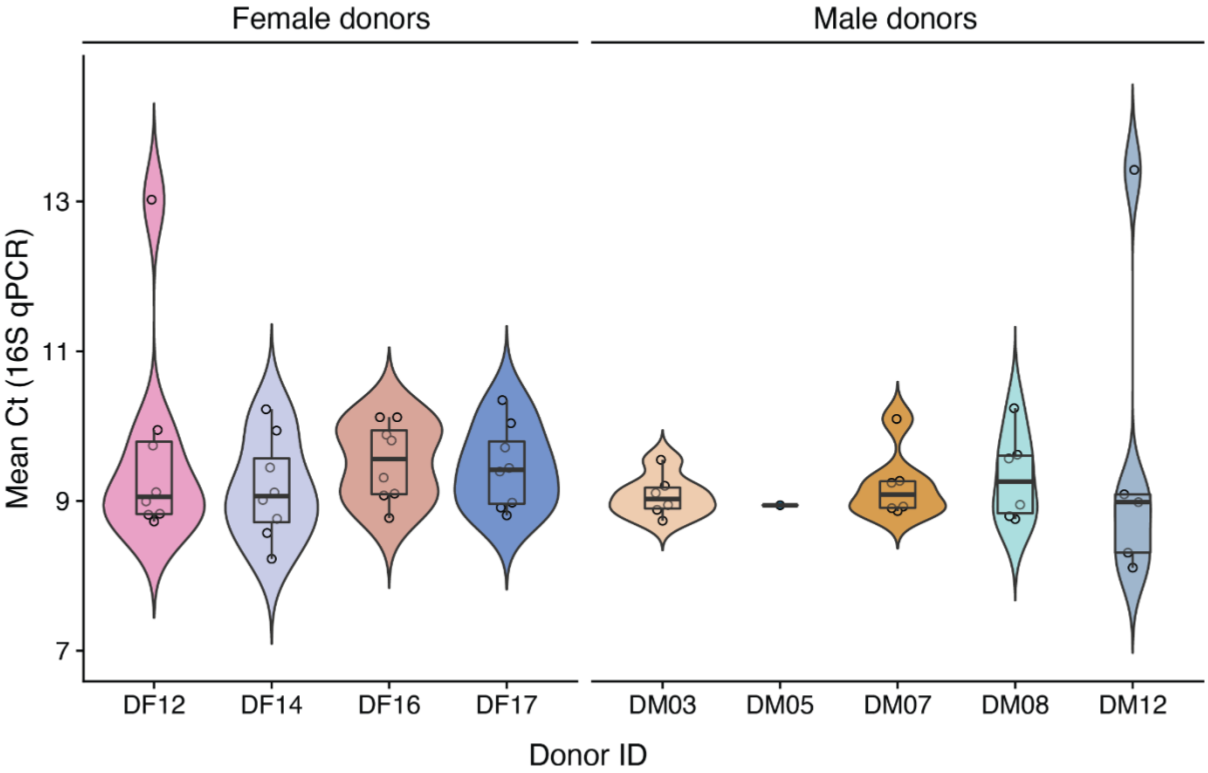

Supplementary Figure 1. Bacterial load of FMT capsules by donor.

Bacterial genome load was determined by quantitative PCR using universal 16S primers (Fwd: 5'-GGTGAATACGTTCCCGG-3'; Rev: 5'-TACGGCTACCTTGTTACGACTT-3'). Female donors donated 8 times (i.e., 8 capsule batches) while male donors donated 6 times (i.e., 6 capsule batches) with the exception of DM05 who was replaced after first batch with DM12. Triplicate qPCR reactions were performed using DNA (1 µl) extracted from the contents of a single donor capsule (from each batch). Reactions were prepared using SYBR Select Master Mix (Applied Biosystems) and performed on a QuantStudio 6 Flex platform (cycling conditions: 50°C 2min, 96°C 2min, then 30 cycles of 95°C 15 sec, 60°C 1min). Each point on the violin plot represents the mean cycling threshold (Ct) value for technical triplicates for each capsule. There was no statistically significant difference in bacterial load between donors (all donors, $p = 0.82$; female donors only, $p = 0.67$; male donors only, $p = 0.93$; Kruskal-Wallis test).

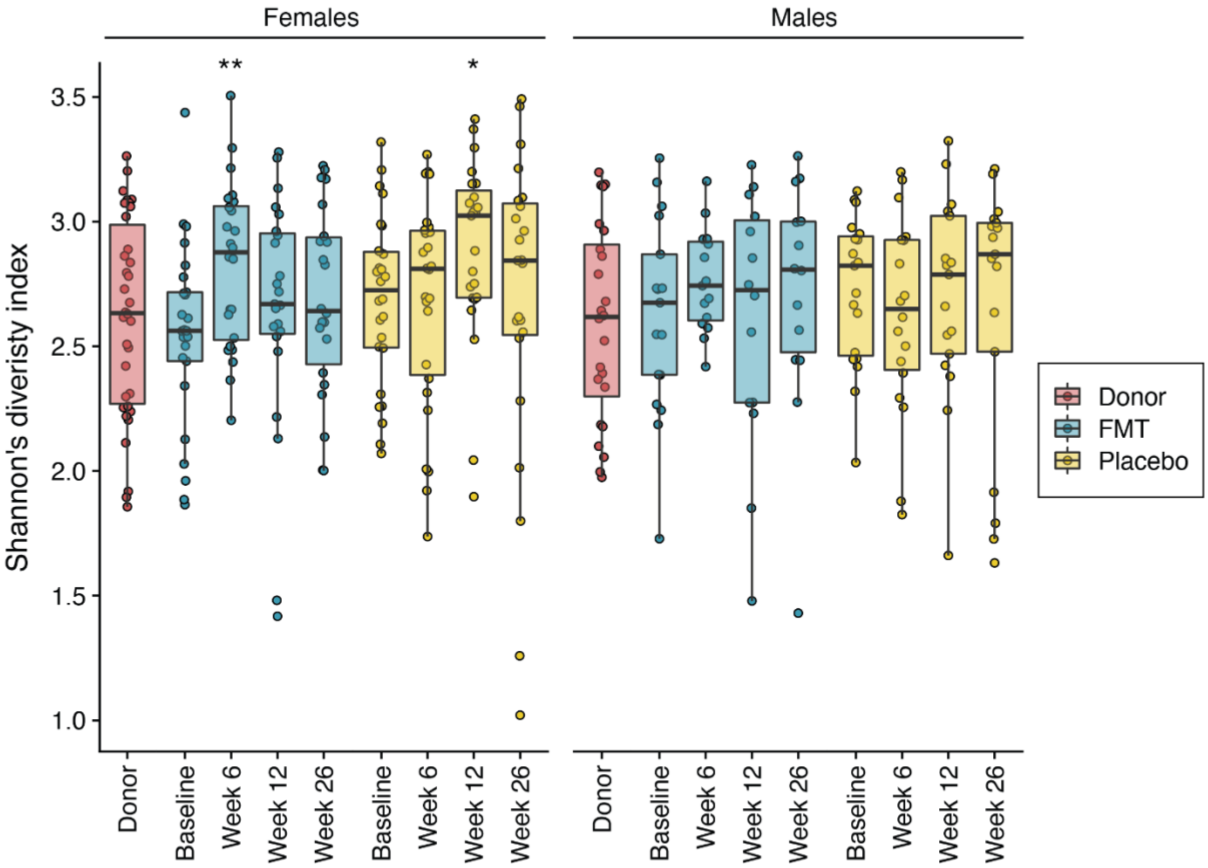

Supplementary Figure 2. Microbial diversity, as measured by Shannon's diversity index, of donors' and recipients' gut microbiomes.

Longitudinal changes in microbial diversity were assessed by Wilcoxon signed-rank test with differences from baseline denoted by *p <0.05, **p <0.005.

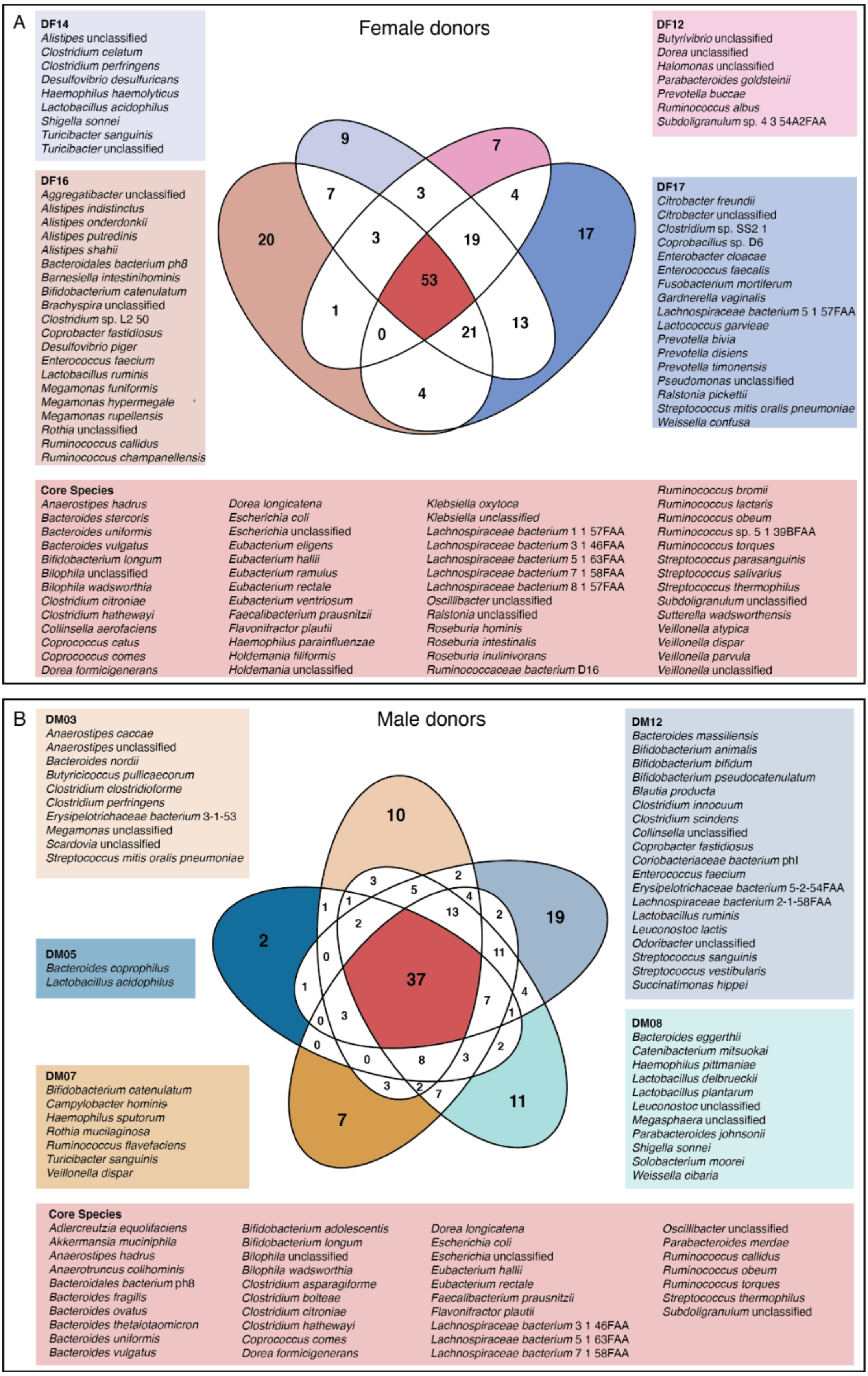

Supplementary Figure 3. Venn diagrams displaying the number of bacterial species in the gut microbiome that were unique to or shared between (A) female donors and (B) male donors.

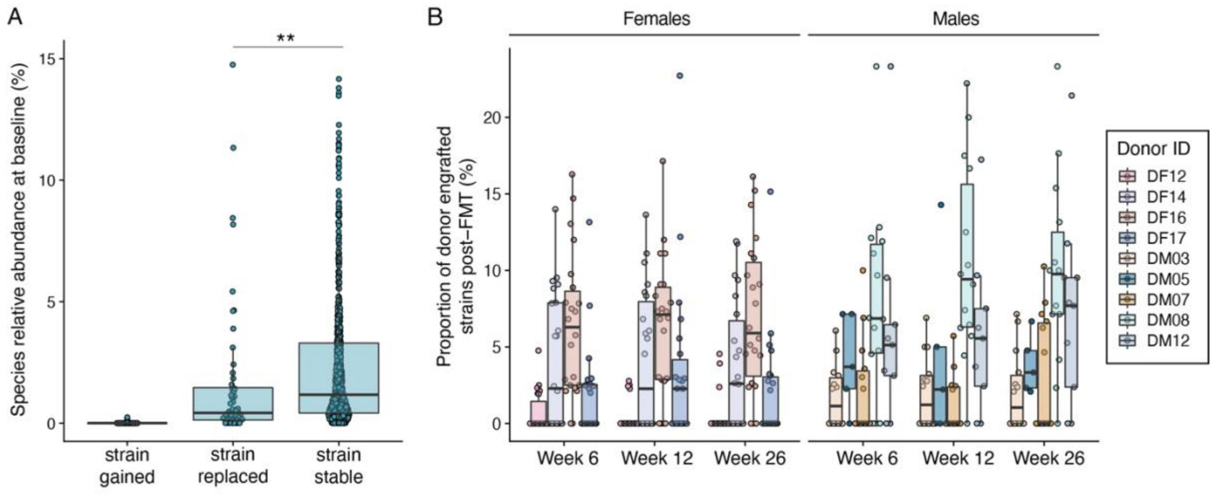

Supplementary Figure 4. Strain engraftment dynamics.

(A) Relative abundance of bacterial species in the gut microbiome at baseline according to their respective dominant strain scenario. Data points represent bacterial species in the gut microbiome of FMT recipients for which strain-level identification was available at baseline and week 6. “Strain gained” represents strains that were acquired at week 6 from species that were not present at baseline. “Strain replaced” represents dominant strains that were replaced with a conspecific donor strain post-FMT. “Strain stable” represents dominant strains that remained stable from baseline to week 6. Wilcoxon rank-sum test was used to compare the difference in relative abundance of species at baseline for strains that were replaced or remained stable. **p <0.005.

(B) Proportion of donor-engrafted bacterial strains in the gut microbiome of FMT recipients at each post-treatment time point. Data points represent FMT recipient metagenome samples which are plotted according to the proportion of strains within their gut microbiome that matched a donor’s strain, subset by donor ID and time point.

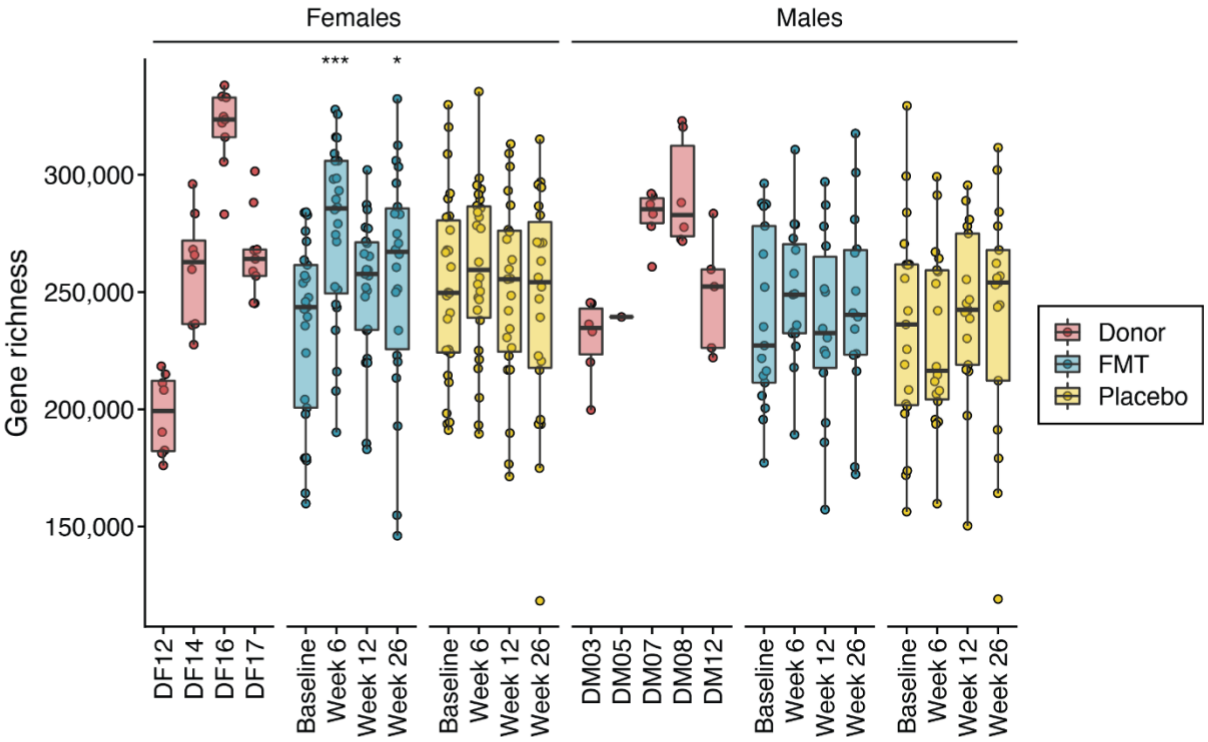

Supplementary Figure 5. Gene richness of donors' and recipients' gut microbiomes.

Longitudinal changes in recipient's gene richness from baseline were assessed by Wilcoxon signed-rank test with significance denoted by *p < 0.05, ***p < 0.0005.
