# Supplementary material for: Strain engraftment competition and functional augmentation in a multi-donor fecal microbiota transplantation trial for obesity
Source: Microbiome. 2021 May 13;9:107. doi: 10.1186/s40168-021-01060-7 (PMC8120839; doi:10.1186/s40168-021-01060-7)
Supplement: Supplementary file 3 — Additional file 2. Supplementary Table 1 [file 40168_2021_1060_MOESM3_ESM.docx]

**Supplementary Table 1.** Variance of bacterial species profiles (Bray-Curtis dissimilarity) explained by each covariate in cross-sectional PERMANOVA.

|  | **Baseline (n = 84)** | | | **Week 6 (n = 82)** | | | **Week 12 (n = 77)** | | | **Week 26 (n = 73)** | | |
| --- | --- | --- | --- | --- | --- | --- | --- | --- | --- | --- | --- | --- |
| **Covariates** | **R^2^** | **p-value** | **q-value** | **R^2^** | **p-value** | **q-value** | **R^2^** | **p-value** | **q-value** | **R^2^** | **p-value** | **q-value** |
| **Sequence batch** | 4·7% | 0·057 | 0·15 | 3·2% | 0·086 | 0·18 | n/a | n/a | n/a | n/a | n/a | n/a |
| **Sex** | 1·3% | 0·31 | 0·40 | 3·7% | 0·001 | **0·013** | 1·3% | 0·37 | 0·46 | 1·7% | 0·18 | 0·28 |
| **Age** | 1·5% | 0·16 | 0·28 | 0·6% | 0·96 | 0·96 | 1·7% | 0·17 | 0·28 | 3·3% | 0·008 | **0·055** |
| **Ethnicity** | 6·5% | 0·032 | 0·14 | 6·2% | 0·050 | 0·15 | 6·9% | 0·060 | 0·15 | 6·0% | 0·22 | 0·31 |
| **Antibiotics** | n/a | n/a | n/a | 1·5% | 0·18 | 0·28 | 1·0% | 0·64 | 0·71 | 1·2% | 0·49 | 0·57 |
| **Treatment group** | 0·6% | 0·95 | 0·96 | 2·7% | 0·006 | **0·055** | 2·9% | 0·011 | **0·060** | 2·2% | 0·063 | 0·15 |

Table contain R^2^ (the proportion of variance explained), nominal p-value based on 10,000 permutation tests, and FDR-adjusted p-values (q-values) after multiple comparisons.

Significant findings are highlighted in bold (q value <0.1).

n/a, not applicable.
