# Supplementary material for: Strain engraftment competition and functional augmentation in a multi-donor fecal microbiota transplantation trial for obesity
Source: Microbiome. 2021 May 13;9:107. doi: 10.1186/s40168-021-01060-7 (PMC8120839; doi:10.1186/s40168-021-01060-7)
Supplement: Supplementary file 4 — Additional file 3. Supplementary Table 2 [file 40168_2021_1060_MOESM4_ESM.docx]

**Supplementary Table 2.** Shifts in the gut microbiome profile of FMT and placebo recipients towards donor microbiomes.

|  |  | **Bray-Curtis Dissimilarity** | | | | **Baseline-adjusted shift** | | | |  |
| --- | --- | --- | --- | --- | --- | --- | --- | --- | --- | --- |
|  |  | **FMT** | | **Placebo** | | **FMT** | | **Placebo** | | **FMT vs. Placebo** |
| **Donor** | **Week** | **N** | **Mean ± SD** | **N** | **Mean ± SD** | **Shift** | **p-value** | **Shift** | **p-value** | **p-value** |
| **DF12** | 0 | 25 | 0.76 ± 0.08 | 26 | 0.78 ± 0.07 |  |  |  |  |  |
|  | 6 | 24 | 0.76 ± 0.09 | 26 | 0.77 ± 0.07 | -0.003 | 0.99 | 0.005 | 0.53 | 0.71 |
|  | 12 | 23 | 0.80 ± 0.07 | 23 | 0.79 ± 0.08 | -0.029 | 0.17 | -0.009 | 0.75 | 0.42 |
|  | 26 | 22 | 0.79 ± 0.08 | 23 | 0.76 ± 0.10 | -0.017 | 0.42 | 0.019 | 0.39 | 0.25 |
| **DF14** | 0 | 25 | 0.70 ± 0.07 | 26 | 0.68 ± 0.10 |  |  |  |  |  |
|  | 6 | 24 | 0.71 ± 0.09 | 26 | 0.69 ± 0.12 | -0.010 | 0.53 | -0.008 | 0.84 | 0.74 |
|  | 12 | 23 | 0.73 ± 0.10 | 23 | 0.71 ± 0.10 | -0.034 | 0.070 | -0.030 | 0.45 | 0.42 |
|  | 26 | 22 | 0.69 ± 0.09 | 23 | 0.68 ± 0.11 | 0.013 | 0.35 | -0.008 | 0.80 | 0.48 |
| **DF16** | 0 | 25 | 0.66 ± 0.10 | 26 | 0.64 ± 0.09 |  |  |  |  |  |
|  | 6 | 24 | 0.58 ± 0.06 | 26 | 0.64 ± 0.10 | 0.082 | **0.0025** | 0.001 | 0.92 | **0.016** |
|  | 12 | 23 | 0.62 ± 0.06 | 23 | 0.64 ± 0.09 | 0.041 | 0.12 | -0.005 | 0.96 | 0.27 |
|  | 26 | 22 | 0.58 ± 0.11 | 23 | 0.65 ± 0.10 | 0.081 | **0.0066** | -0.016 | 0.54 | **0.0031** |
| **DF17** | 0 | 25 | 0.68 ± 0.09 | 26 | 0.69 ± 0.08 |  |  |  |  |  |
|  | 6 | 24 | 0.70 ± 0.07 | 26 | 0.69 ± 0.07 | -0.024 | 0.56 | -0.004 | 0.67 | 0.93 |
|  | 12 | 23 | 0.72 ± 0.08 | 23 | 0.67 ± 0.07 | -0.037 | 0.092 | 0.013 | 0.41 | 0.061 |
|  | 26 | 22 | 0.70 ± 0.06 | 23 | 0.69 ± 0.08 | -0.022 | 0.54 | -0.004 | 0.64 | 0.96 |
| **DM03** | 0 | 17 | 0.65 ± 0.12 | 19 | 0.64 ± 0.09 |  |  |  |  |  |
|  | 6 | 15 | 0.69 ± 0.11 | 18 | 0.64 ± 0.10 | -0.050 | 0.30 | -0.009 | 0.64 | 0.51 |
|  | 12 | 14 | 0.67 ± 0.12 | 17 | 0.68 ± 0.10 | -0.024 | 0.63 | -0.047 | 0.11 | 0.60 |
|  | 26 | 14 | 0.64 ± 0.10 | 17 | 0.67 ± 0.12 | 0.004 | 0.86 | -0.041 | 0.13 | 0.17 |
| **DM05** | 0 | 6 | 0.77 ± 0.08 | 5 | 0.71 ± 0.11 |  |  |  |  |  |
|  | 6 | 5 | 0.79 ± 0.04 | 5 | 0.76 ± 0.05 | 0.001 | 0.81 | -0.052 | 0.44 | 0.42 |
|  | 12 | 5 | 0.76 ± 0.10 | 5 | 0.68 ± 0.14 | 0.039 | 0.63 | 0.027 | 0.31 | 0.84 |
|  | 26 | 5 | 0.81 ± 0.05 | 5 | 0.75 ± 0.13 | -0.017 | 1.00 | -0.044 | 0.63 | 0.84 |
| **DM07** | 0 | 17 | 0.59 ± 0.15 | 19 | 0.59 ± 0.10 |  |  |  |  |  |
|  | 6 | 15 | 0.62 ± 0.12 | 18 | 0.63 ± 0.12 | -0.061 | 0.095 | -0.035 | 0.26 | 0.53 |
|  | 12 | 14 | 0.61 ± 0.13 | 17 | 0.66 ± 0.10 | -0.035 | 0.22 | -0.060 | **0.04** | 0.60 |
|  | 26 | 14 | 0.57 ± 0.11 | 17 | 0.67 ± 0.14 | 0.003 | 0.90 | -0.074 | 0.07 | 0.28 |
| **DM08** | 0 | 17 | 0.61 ± 0.10 | 19 | 0.58 ± 0.08 |  |  |  |  |  |
|  | 6 | 15 | 0.61 ± 0.10 | 18 | 0.61 ± 0.08 | -0.010 | 0.72 | -0.033 | 0.20 | 0.66 |
|  | 12 | 14 | 0.57 ± 0.12 | 17 | 0.59 ± 0.10 | 0.040 | 0.14 | -0.019 | 0.61 | 0.059 |
|  | 26 | 14 | 0.58 ± 0.08 | 17 | 0.64 ± 0.11 | 0.033 | 0.43 | -0.064 | 0.06 | **0.044** |
| **DM12** | 0 | 11 | 0.65 ± 0.13 | 14 | 0.66 ± 0.12 |  |  |  |  |  |
|  | 6 | 10 | 0.69 ± 0.12 | 13 | 0.71 ± 0.08 | -0.057 | 0.13 | -0.048 | 0.34 | 0.65 |
|  | 12 | 9 | 0.61 ± 0.17 | 12 | 0.73 ± 0.07 | 0.035 | 0.73 | -0.044 | 0.18 | 0.22 |
|  | 26 | 9 | 0.62 ± 0.11 | 12 | 0.73 ± 0.11 | 0.024 | 0.43 | -0.051 | 0.23 | 0.17 |

Shifts in gut microbiome measured by Bray-Curtis dissimilarity and adjusted for baseline dissimilarity values.

N represents the number of participants included in each treatment group at each time point.

Within-group differences in baseline-adjusted shifts towards donor profiles at each time point were assessed by Wilcoxon signed-rank tests.

The difference in baseline-adjusted shifts towards donor profiles between FMT and placebo groups were assessed by Wilcoxon rank-sum tests.

Significant findings are highlighted in bold (p value < 0.05).
