# Supplementary material for: Strain engraftment competition and functional augmentation in a multi-donor fecal microbiota transplantation trial for obesity
Source: Microbiome. 2021 May 13;9:107. doi: 10.1186/s40168-021-01060-7 (PMC8120839; doi:10.1186/s40168-021-01060-7)
Supplement: Supplementary file 5 — Additional file 4. Supplementary Table 3 [file 40168_2021_1060_MOESM5_ESM.docx]

**Supplementary Table 3.** MetaCyc pathways that were differentially abundant between FMT and placebo recipients’ gut microbiomes.

| **Time point** | **MetaCyc pathway** | **Model coefficient** | **stderr** | **N** | **N not 0** | **p-value** | **q-value** |
| --- | --- | --- | --- | --- | --- | --- | --- |
| Week 6 | PANTOSYN-PWY: pantothenate and coenzyme A biosynthesis I | 60.5 | 14.4 | 79 | 79 | 6.8E-05 | 0.0081 |
| **Week 6** | **PYRIDNUCSYN-PWY: NAD biosynthesis I (from aspartate)** | **-55.6** | **14.8** | **79** | **79** | **3.4E-04** | **0.0307** |
| Week 6 | COA-PWY: coenzyme A biosynthesis I | 53.3 | 16.1 | 79 | 79 | 1.4E-03 | 0.1036 |
| **Week 6** | **PWY-2941: L-lysine biosynthesis II** | **-60.6** | **19.5** | **79** | **79** | **2.6E-03** | **0.1233** |
| Week 6 | PWY-3841: folate transformations II | 60.8 | 19.4 | 79 | 79 | 2.5E-03 | 0.1233 |
| Week 6 | PWY0-1298: superpathway of pyrimidine deoxyribonucleosides degradation | 13.3 | 4.3 | 79 | 79 | 2.7E-03 | 0.1233 |
| **Week 6** | **POLYAMSYN-PWY: superpathway of polyamine biosynthesis I** | **-29.3** | **9.7** | **79** | **79** | **3.4E-03** | **0.1374** |
| **Week 6** | **PWY-5189: tetrapyrrole biosynthesis II (from glycine)** | **-14.7** | **5.1** | **79** | **77** | **4.9E-03** | **0.1730** |
| **Week 6** | **PWY-7371: 1,4-dihydroxy-6-naphthoate biosynthesis II** | **-15.8** | **5.5** | **79** | **62** | **5.3E-03** | **0.1730** |
| Week 6 | PWY-6385: peptidoglycan biosynthesis III (mycobacteria) | 58.4 | 20.7 | 79 | 79 | 6.2E-03 | 0.1843 |
| Week 12 | COLANSYN-PWY: colanic acid building blocks biosynthesis | 20.7 | 6.3 | 73 | 73 | 1.6E-03 | 0.1835 |
| Week 26 | COLANSYN-PWY: colanic acid building blocks biosynthesis | 27.2 | 5.8 | 73 | 72 | 1.2E-05 | 0.0015 |
| Week 26 | PWY-7323: superpathway of GDP-mannose-derived O-antigen building blocks biosynthesis | 21.5 | 4.5 | 73 | 72 | 1.0E-05 | 0.0015 |
| Week 26 | ARGININE-SYN4-PWY: L-ornithine de novo biosynthesis | 51.3 | 11.3 | 73 | 73 | 2.3E-05 | 0.0018 |
| Week 26 | PYRIDOXSYN-PWY: pyridoxal 5’-phosphate biosynthesis I | 48.4 | 10.9 | 73 | 73 | 3.4E-05 | 0.0020 |
| Week 26 | PWY0-845: superpathway of pyridoxal 5’-phosphate biosynthesis and salvage | 57.9 | 13.3 | 73 | 73 | 4.4E-05 | 0.0022 |
| Week 26 | 1CMET2-PWY: *N*^10^-formyl-tetrahydrofolate biosynthesis | 65.5 | 15.7 | 73 | 73 | 8.1E-05 | 0.0034 |
| Week 26 | PWY-5101: L-isoleucine biosynthesis II | 31.9 | 7.7 | 73 | 73 | 9.7E-05 | 0.0034 |
| Week 26 | PWY-6168: flavin biosynthesis III (fungi) | 62.0 | 14.9 | 73 | 73 | 8.9E-05 | 0.0034 |
| Week 26 | PWY-7282: 4-amino-2-methyl-5-phosphomethylpyrimidine biosynthesis (yeast) | 64.5 | 17.0 | 73 | 73 | 3.1E-04 | 0.0100 |
| Week 26 | PWY-3841: folate transformations II | 68.3 | 19.6 | 73 | 73 | 8.5E-04 | 0.0231 |
| Week 26 | PWY-6531: mannitol cycle | 6.0 | 1.7 | 73 | 71 | 8.1E-04 | 0.0231 |
| Week 26 | PWY-6703: preQ0 biosynthesis | 60.7 | 17.9 | 73 | 73 | 1.1E-03 | 0.0288 |
| Week 26 | PWY-5154: L-arginine biosynthesis III (via *N*-acetyl-L-citrulline) | 23.7 | 7.6 | 73 | 73 | 2.7E-03 | 0.0643 |
| Week 26 | ANAGLYCOLYSIS-PWY: glycolysis III (from glucose) | 57.0 | 20.4 | 73 | 73 | 6.6E-03 | 0.1467 |

MetaCyc pathways that were found to be differentially abundant between FMT and placebo recipient gut microbiomes at each specified time point by MaAsLin2 (significance q<0.2).

N represents the number of participant samples included in the model.

N not 0 represents the number of participant samples where the specified pathway had an abundance count > 0.

Rows in bold represent pathways that were enriched in FMT recipients.

Nominal p-values were adjusted for multiple testing using Benjamini-Hochberg procedure to obtain q-values.
